# Advanced Compliant Anti-Gravity Robot System for Lumbar Stabilization Exercise Using Series Elastic Actuator

**DOI:** 10.1109/JTEHM.2021.3135974

**Published:** 2021-12-15

**Authors:** Joowan Kim, Wonje Choi, Sungmoon Hur, Jaeheung Park

**Affiliations:** Graduate School of Convergence Science and TechnologySeoul National University 26725 Gwanak-gu Seoul 08826 Republic of Korea; Samsung Advanced Institute of Technology 65473 Yeongtong-gu Suwon-si 16677 Republic of Korea; Graduate School of Convergence Science and Technology, ASRISeoul National University 26725 Gwanak-gu Seoul 08826 Republic of Korea; Advanced Institute of Convergence Technology Suwon-si 16229 Republic of Korea

**Keywords:** Lumbar stabilization exercise, rehabilitation robot, surface electromyography, series elastic actuator

## Abstract

*Background*: The lumbar stabilization exercise is one of the most recommended treatments in medical professionals for patients suffering from low back pain. However, because lumbar stabilization exercise is calisthenics, it is challenging to perform because of the body load of the elderly, disabled, and patients that lack muscle strength. Additionally, it interferes with the effect of exercise because it can strain parts of the body. *Methods*: To overcome them, a compliant anti-gravity rehabilitation proto-type device using the Series Elastic Actuator (SEA) was developed previously to provide quantitative assist force to the person, producing similar exercise effects with calisthenics. From an exercise experiment with 20 participants, it caused discomfort to participants during exercise owing to the non-ergonomic design of the previous device. Different muscle activation tendencies were observed between calisthenics and exercise using the device. For advanced technical solutions to clinical needs, which is exercise using the rehabilitation robot to produce a similar effect to calisthenics, the mechanical design of the rehabilitation robot was improved based on the previous device after receiving feedback from clinical trials and static analysis. For the safety of exercise using the robot, a cascade PID-PI controller was used to reduce the influence of friction and disturbance due to the external movement. *Results*: Surface electromyography(sEMG) signal from lumbar muscles showed desired monotonic reduction ratio and higher similarity results compared to the previous device, which proved the exercise effectiveness using the robot. *Conclusion*: The proposed robot is considered as a solution to a clinical need of lumbar rehabilitation for the elderly, disabled, and patients.

## I. Introduction

### A. Overview of Low Back Pain

Low back pain (LBP) occurs in the lower part of the spine. It is referred to as the lumbar part from the 12th rib to the pelvis, and includes five lumbar bones and the first sacrum. Low back pain is a highly prevalent disease worldwide. Approximately 70 to 80% of the population suffers from the disease; there is a high prevalence from 35 to 80 years old [1]. The causes of LBP have been reported to be affected by various factors such as psychogenic, neurogenic, spinal, vascular, and visceral disease [2]. Additionally, LBP also occurs because of trauma, tumors, inflammation, and disc prolapse [3].

However, the major problem of LBP lies in the inability to accurately diagnose the cause of the disease [4]. 85% of LBP is a so-called non-specific LBP that does not have neurological symptoms or other severe diseases and is hard to diagnose through imaging [5]. This non-specific LBP presents difficulties in diagnostic treatment. LBP is divided into acute, subacute, and chronic, depending on the duration of the pain. If it develops to chronic, the likelihood of experiencing chronic complications other than LBP increases and may develop into intractability due to chronicity. Consequently, when back pain occurs, appropriate treatment should be performed.

### B. Treatment and Exercise Therapy of LBP

The treatment of LBP is primarily divided into surgical and non-surgical treatment. For most pain, medical professionals recommend non-surgical treatment [6]. Non-surgical treatment is divided into drug treatment and exercise therapy; drug treatment aims to relieve pain through the injection of drugs such as steroids and pain relievers. Because drug treatment has a temporary pain-reducing effect, exercise therapy is the most recommended method as a guideline as because it not only relieves pain but also prevents recurrence and improves lumbar motor function [7], [8].

Effective exercise therapy for LBP has been proposed in many studies in the rehabilitation medical field. Typical proposed exercises include the McKenzie extension exercise [9], Williams flexion exercise [10], and the lumbar stabilization exercise [11]. Among them, the lumbar stabilization exercise aims to train the muscles around the spine and is designed based on epidemiological analysis and clinical verification. Therefore, it has the effect of reducing pain and improving functions within a relatively short time [12]. The lumbar muscles used in the lumbar stabilization exercise consist of a local and global muscular system [13]. The local muscular system is directly connected to the spine and acts against lumbar movement and gravity. The global muscular system is used when generating large torques to the spine. Stabilization exercises aim to train both of these muscle groups. The lumbar stabilization exercise has the advantage of improving muscle strength and restoring the control ability of the patient’s lumbar region through exercise training. A specific muscle can be activated through various postures and maintain a constant posture to a pain-free range.

### C. Limitations of Lumbar Stabilization Exercise

Although lumbar stabilization exercise has a therapeutic effect, there are some weaknesses for all patients to adopt. The first problem is the load generated on the patient’s body during exercise, making it challenging for patients with a lack of muscle strength to perform the exercise. The load on the body is divided into loads of the back muscles and loads other than the back muscles. The lumbar stabilization exercise is calisthenics, thus, these two loads coexist. In other words, the pain caused by the loads other than the back muscles makes it difficult for patients with LBP to perform the exercise correctly. Besides, unlike a healthy person, LBP patients sometimes give up training because of weak spinal stability and lumbar muscle strength. To perform the exercise within the pain-free range, gradual training is required to suit the patient’s lumbar condition.

In a previous study, a proto-type of a rehabilitation device for LBP was proposed to overcome the difficulties of the lumbar stabilization exercise [14]. The device was designed to meet two requirements: first, to control the load on the back muscles quantitatively, and second, to lower the loads other than the back muscles during exercise using the device. As a preliminary experiment of the device, 20 healthy subjects participated in the exercise evaluation using the sEMG sensor. From the experiment, some exercises using the device had similarities to muscle activation with calisthenics. However, in specific postural exercises, the results showed low similarity to muscle activation. Besides, some subjects complained of difficulties in balancing in the exercise using the device. The experiment results showed that the design goal and clinical needs of the device were not fully achieved. Consequently, the above-described problem occurs when a person performs the lumbar stabilization exercise using a device. The arrangement of the support and assist force points of the device is not appropriate compared to calisthenics. As a result of misalignment, the load of the target muscle cannot be quantitatively controlled. Additionally, the muscles that do not require exercise are activated during exercise.

This study proposes an improved rehabilitation robot system appropriate for lumbar stabilization exercises, by complementing the limitations and disadvantages of the previous rehabilitation device. To produce a similar exercise effect to that of calisthenics of lumbar stabilization exercise, the mechanical design of the hardware system excluding the actuator was modified to apply the ergonomic design through clinical feedback and static analysis received from the previous experiment. To reduce the impedance when operating the robot, the Cascade PID-PI controller, which is robust to disturbance and friction, which can also accurately follow the desired force, was applied to the system to improve the control performance. Additionally, fifteen healthy subjects participated in the experiment to evaluate the lumbar stabilization exercise using the robot and prove the exercise effects through the similarity analysis of sEMG signals from target muscles.

The paper is organized as follows. In Chapter 2, we describe the Big three exercise, the target exercise, which is a representative stabilization exercise, and propose the design goal of a robot system for the spinal rehabilitation exercise. The concept, detailed design of the robot, and hardware specifications including the actuator and its controller are also described. Chapter 3 presents the experiment method of the exercise using the robot, experiment results of the sEMG signal, and analysis of the experiment results through participants. The paper is concluded in Chapter 4 with discussion and future works.

## II. Proposed Robot System for Lumbar Stabilization Exercise

### A. Big Three Lumbar Stabilization Exercise

**FIGURE 1.**
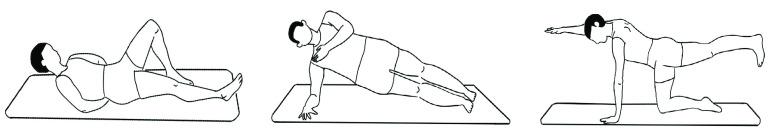
Big three lumbar stabilization exercise (curl-up, side-bridge, bird-dog).

Various postures have been proposed for the lumbar stabilization exercise. In this study, McGill’s big three exercise, whose therapeutic effect had been verified sufficiently was selected [15]. Another reason for choosing this exercise is that clinical research results substantiated that it can train a specific back muscle group for each exercise posture. The big three exercise consists of three postures: curl-up, side-bridge, and bird-dog. These exercises aim to train five lower back muscles: Rectus abdominis (RA), external oblique (EO), internal oblique (IO), thoracic erector spinae (TES), and lumbar erector spinae (LES). These muscle groups are involved in the trunk flexion and extension of the lower back, lateral flexion, and rotational movements of the lumbar. Muscles related to lower back movements are summarized and marked in the [Table 1]. The posture of the Big Three exercise is as follows [Fig. 1]:

**TABLE 1:** Target Muscles of Lumbar Rehabilitation

Muscle	Rectus abdominis (RA)	External oblique (EO)	Internal oblique (IO)	Thoracic erector spinae (TE)	Lumar Erector Spinae (LE)
Related Motion	Trunk flexion	Trunk flexion, Lateral flexion, Trunk rotation	Trunk flexion, Lateral flexion, Trunk rotation	Trunk extension	Lateral flexion, Trunk extension
Location

#### 1) Curl-Up

The motion of lying down with the back on the floor, placing hands behind the back, bending one knee, and bending the upper body with the thoracic spine as a rotation axis. Shoulders are slightly off the floor during the exercise. The curl-up exercise produces the exercise effect of lumbar flexion, activating the RA, EO, and IO muscle groups. Because this exercise requires maintaining the posture of the cervical spine, it may cause an overload on the neck, causing difficulties in performing the exercise for patients.

#### 2) Side-Bridge

The person lies on one side and then supports the body with one foot and elbow to make it stand to the side. This exercise uses the EO, and IO, and LE muscle groups for lateral flexion of the lower back. However, it is challenging to perform the exercise for patients with upper limb disease owing to the load on the elbow and arm muscles.

#### 3) Bird-Dog

Here, one arm and the other side knee are supported on the ground, and the opposite arm and leg are kept parallel to the ground. Bird-dog is an exercise in which trunk extension and rotation are performed simultaneously. The EO, IO, TE, and LE muscle groups are activated. This exercise requires the use of the upper and lower limbs and is a strenuous exercise for patients with weak muscle strength.

### B. Design Goals of Rehabilitation Robot

**FIGURE 2.**
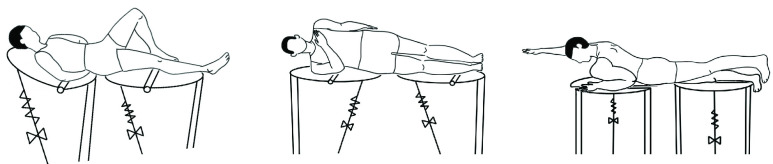
Big three lumbar stabilization exercise using rehabilitation robot.

The design goals of the rehabilitation robot for the lumbar stabilization exercise are as follows: First, the target muscles of the big three exercises are trained during the exercise using the rehabilitation robot [Fig. 2]. In other words, when a specific posture exercise is performed using the robot, the mainly used muscle pattern is the same as calisthenics. Second, it is possible to quantitatively control the load on the lower back muscles to be trained, and simultaneously, reduce the load other than the lower back muscles. When the patient is in a posture for lumbar stabilization, the robot is designed with a structure that applies a precise force in the opposite direction of gravity to reduce the vertical load applied to the spine and allow quantitative load adjustment. Additionally, a series elastic actuator was applied to ensure safety in the interaction between the robot and human to perform accurate force control output.

### C. Conceptual and Mechanical Design of the Robot

**FIGURE 3.**
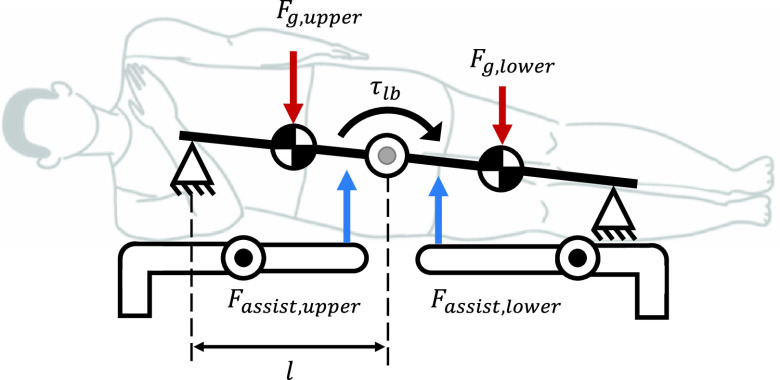
Dynamics of lumbar stabilization exercise using rehabilitation robot.

The proposed robotic system was designed as a bed type by analyzing the lumbar stabilization motion with a static model consisting of a support point and a mass. The reason for the bed-type approach is that the external force (e.g. gravity) applied to the spine is minimized when a person is lying down. This method minimizes the spinal burden on the patient to perform lumbar exercise relatively safely. The patient lies down on the robot for the lumbar stabilization exercise. The support point is designed to be adjustable from 1360 mm to a maximum of 1650 mm so that adult males can perform exercise adequately. Each bed module can be translated, rotated, and fixed, so the position of the actuator can be adjusted according to the patient’s body size and posture. The robot is composed of two anti-gravity bed modules, which are composed of low rigidity series elastic actuators (SEA). The assist force of the SEA applies a force to the opposite direction of gravity in the mass part to quantitatively adjust the patient’s load. Additionally, just as the ground plays the role of the contact and support points when performing calisthenics, a part of the bed is configured as a fixed type to provide a support point. From the perspective of each bed module, it provides one assist force point and one support point. Considering the side-bridge as an example, it can be modeled as follows [Fig. 3]:

\begin{equation*} \tau _{lb} =\left ({{1-\alpha } }\right)\cdot l\cdot F_{g,human}\tag{1}\end{equation*}

In equation (1), $\tau _{lb} $ is the torque applied to the lumbar, $l$ is the length between the support and mass points on the waist, $F_{g,human} $ is human gravity, and $F_{assist} $ is the assist force of the actuator. Because the assist force is applied as the assist ratio $\alpha $ relative to human gravity, equation (1) can be converted as follows:

\begin{equation*} \tau _{lb} =l\cdot \left ({{F_{g,human} -F_{assist}} }\right)\tag{2}\end{equation*}

Because the support plate of the robot bed and the mass of the mattress are also present, the corresponding force is removed by gravity compensation, and the load applied to the lumbar is quantitatively adjusted using Equation (2), enabling the patient to perform the exercise using the robot.

For the mechanism design changed from the previous prototype, the support point, mass, and axis of rotation were derived as shown in the figure through the feedback and static analysis of 20 experimental participants, and the improved components are primarily organized into three parts [Table 2]:

**TABLE 2:** Comparison for Static Dynamic Analysis of Lumbar Exercise With or Without Rehabilitation Robot

Exercise	Curl-up			Side-bridge			Bird-dog		
Static Analysis									
Method	Calisthenics	Previous Device	Advanced Robot	Calisthenics	Previous Device	Advanced Robot	Calisthenics	Previous Device	Advanced Robot

First, the robot bed size was reduced to fit the average body size. The bed in the previous device was circular with a diameter of 720 mm. However, after conducting the experiment in the previous exercise evaluation, it was difficult to maintain the limbs’ posture in the exercise using a robot because the bed size was too large for the exercise. Accordingly, the bed size was reduced to 600 mm.

Second, the support parts fixed to both ends of the robot were arranged by dividing the bed modules into 1:1 and 3:1. This is to make the exercise effects of the calisthenics and the exercise using the robot similar. In the previously developed device, the bed was composed of a single circular module, thus, the entire bed rotated when the actuator was driven. Unlike the calisthenics that supported the ground with arms and legs, there was no support point. As shown in the static analysis [Table 2], the rotation axis of the lumbar spine of the previous device in the curl-up was different from that of the calisthenics owing to misalignment of the support point. Side-bridge and bird-dog were also different from the rotation axis of the calisthenics owing to the absense of the support point of the previous device. The result of misalignment that causes the inconsistent muscle activity between the exercise using the robot and calisthenics was obtained through experiments. In addition, from the survey after the experiment, 50% of all the 20 participants had difficulty maintaining posture owing to the lumbar rotation in the curl-up, 20% of the participants complained of no cervical vertebral fixation in the side-bridge, and 55% complained of difficulty in balancing in the bird-dog owing to no support point. The absence of the support point can make it challenging to perform the exercise using the robot. Moreover, inconsistent results of target muscles activation were not the results we are aiming for. For this reason, the bed modules were divided into 1:1 and 3:1 to place the support point on the robot, respectively.

**FIGURE 4.**
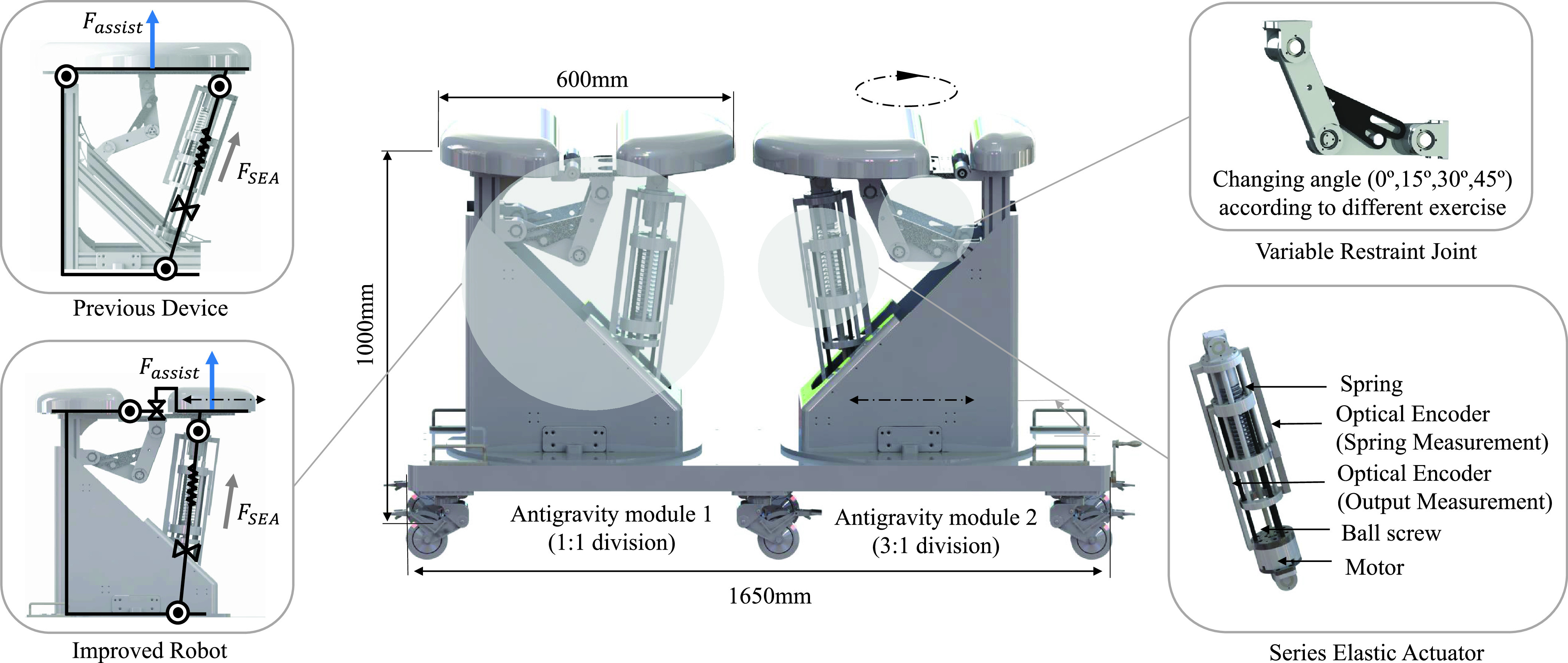
Conceptual design of rehabilitation robot for lumbar stabilization exercise.

Third, by adding a sliding joint to the bed where the assist force is applied, the joint position of the robot is diversified through linear motion (0 to 100 mm). Through this, the position of the assist force point can be changed. In the previous study, the bird-dog posture showed a low similarity of 80% in the similarity analysis with the calisthenics. This was the result of an experiment that caused by the position of the assist force point, provided the assist force did not coincide with the positions of one arm and the other leg in the calisthenics. Besides, in the curl-up posture, the joint movement exists in the region close to the thoracic spine. However, the previously developed device exists in part close to the lumbar spine because the position of the assist force point cannot be changed. Therefore, the robot was designed to change the acting point of the force’s position by adding a sliding joint to the bed of the assist force [Fig. 4].

### D. Actuator and Restraint Joint

The actuator for the robot must meet two conditions: First, it must generate a large force that can sufficiently support a person’s weight and produce a precise force that provides the assist force quantitatively when interacting with a person. Second, for a robot that interacts with humans, safety must be secured through low impedance and adaptive characteristics. To satisfy these conditions, the SEA was used in this study [16]. The SEA is an actuator that takes advantage of the low elasticity material connected in series between the load and motor [17]. The designed actuator is a unidirectional SEA, and the one used in the previous prototype was applied. The SEA can output a force of up to 1570 N and support up to 120 kg weight.

The principle of the actuator involves converting the rotational motion of the electric motor into translational motion through a ball screw; the ball screw controls the displacement of the spring [Fig. 4]. To implement the force measurement through SEA, optical encoders were used to measure the position of the spring input end and the output end to obtain the spring deformation. Finally, the force can be measured through the spring deformation. The resolution of the encoder enables the force measurement with an accuracy of 0.79 mN. The SEA is controlled by a Twincat-based PC (i5, 16G RAM) for real-time control and communicates with a 1 kHz control cycle through an Ethercat bridge. The specifications of the SEA used in rehabilitation robots are summarized in the table below [Table 3].

**TABLE 3:** The Specification of the SEA

Device	Specification	Value
SEA	Maximum force(N)	1570
	Rated force (N)	1200
	Maximum Power (W)	430
	Maximum Stroke (mm)	273
	Maximum Speed (mm/sec)	241.67
Ball screw	Lead (mm)	5
Spring	Stiffness (N/mm)	15.7
	Length (mm)	250
	Maximum Deformation (mm)	100

Two features are required to perform lumbar stabilization exercises on the rehabilitation robot. First, a rigid body mode that supports the patient to board the robot and take a posture safely is needed. It is difficult to implement this using a low elasticity SEA only; therefore, we add a variable restraint joint that can limit the robot’s range of motion. When the SEA output the maximum rated force, the restraint joint can fix the robot’s motion range horizontally or at a specific angle (0°, 15°, 30°, 45°) [Fig. 4].

The second feature is to measure human gravity to calculate the quantitative assist force of the robot. After a person boards the robot in the initial rigid mode, $F_{assist} $ is the gradually reduced force from the maximum force to when a certain small displacement occurs (based on 3 mm). $F_{assist} $ at this moment is set as the approximate value of human gravity. After performing these two steps in advance, the robot’s anti-gravity force is applied quantitatively to the aboard patient by sequentially reducing the assistance rate for human gravity (70 to 100%) to provide the lumbar stabilization exercise using the robot.

### E. Controller of Sea

**FIGURE 5.**
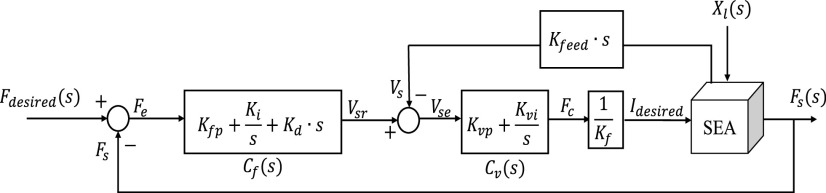
Block diagram of cascade PID-PI controller. ( $F_{desired} $: desired force, $C_{f} $: main controller with force feedback, $V_{sr} $: reference velocity, $V_{s} $: velocity of spring deformation, $C_{v} $: 2nd controller with velocity feedback, $F_{c} $: motor force, Idesired: desired current, $X_{l} $: human motion, $F_{s} $: output force.

**FIGURE 6.**
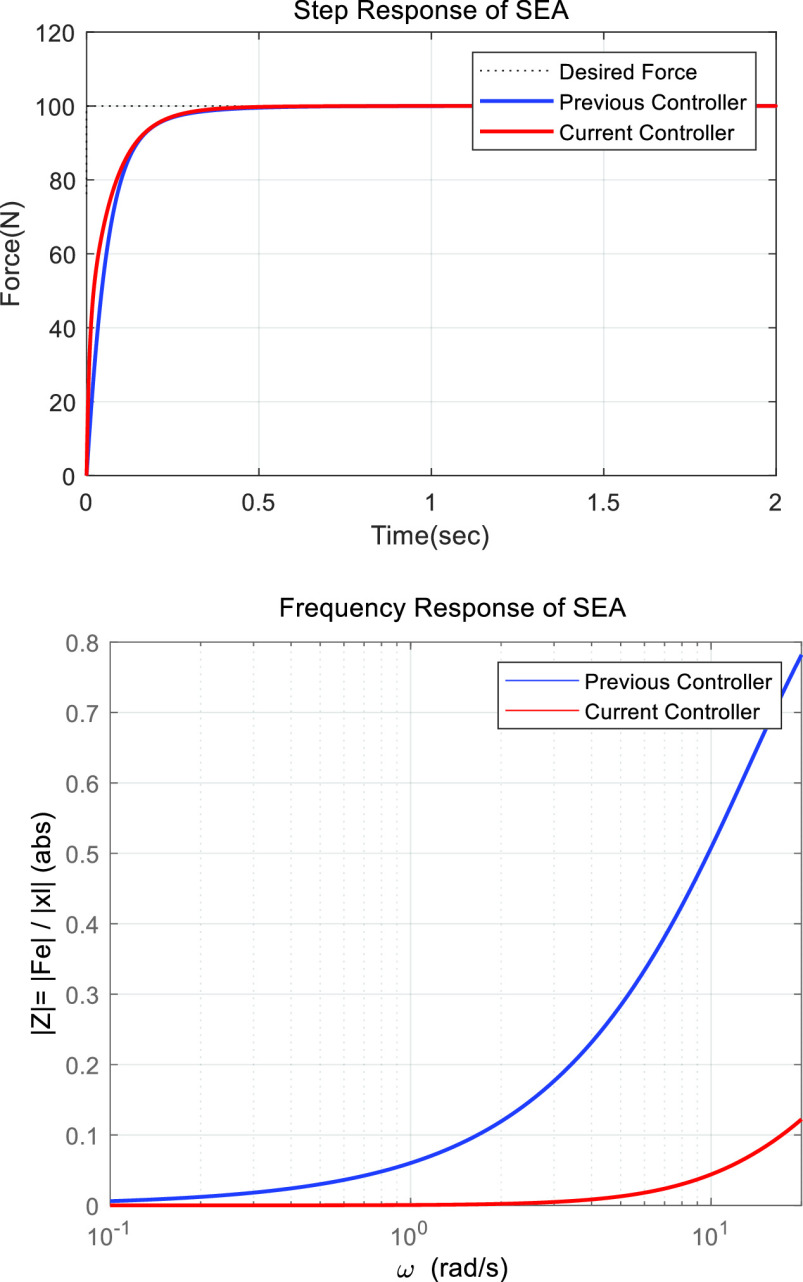
Step and frequency responses of SEA.

The characteristic of this robotic system is that it interacts with humans, and the final output of the controller is force. The output force error of the SEA is influenced by the human motion displacement $X_{l} $. Therefore, it is necessary to apply a controller that is capable of implementing an accurate assist force of the actuator and low impedance characteristic [18]. Additionally, because the motion of the lumbar is in a low-frequency band, it does not require a quick response from the controller. In this experiment, the PID-PI controller was adopted based on the controller performance comparison experiment from the previous research. To reduce the impedance of the robot, a PID-PI cascade controller with a spring deformation rate as feedback (CSRF) was adopted instead of the previous controller [Fig. 5]. The CSRF controller is composed of the main controller $C_{f} \left ({s }\right)$ that handles the force feedback error for the output force, and a secondary controller $C_{v} \left ({s }\right)$ that reduces the error by receiving the velocity of the spring deformation. For the tuning of the gain parameters, the values were obtained using a genetic algorithm based on pre-tuned parameters [19]. In the experiment of the step (desire force 100 N) and frequency responses (0.1~20 rad/sec) to disturbance, the current controller has a similar step response performance compared to the previous controller, but in the frequency response, the magnitude of the impedance is decreased overall between the target frequency [Fig. 6]. The performance of the designed controller was achieved as the goal of the robot that the impedance was reduced and constantly regulating force even in disturbances caused by human movement.

## III. Experiment and Results

This chapter presents the experiment of rehabilitation exercise using the advanced robot and an sEMG signal. The experiment method using the robot is explained in III-A, and the pre-processing method and data acquisition of the sEMG sensor used in the experiment are explained. Based on the acquired sEMG data through experiment, III-B conducts the Mann-Whitney U test, a statistical method, to check whether the calisthenics and the exercise using the robot train the same target muscle. In addition, the characteristics for each exercise are indicated through sEMG bar graph analysis. The comparison of exercise effect between the previous device and the advanced robot is described in III-C. Through similarity analysis, we analyze how similar the exercise using the two devices is to the calisthenics with confusion matrics.

### A. Experiment Method

**FIGURE 7.**
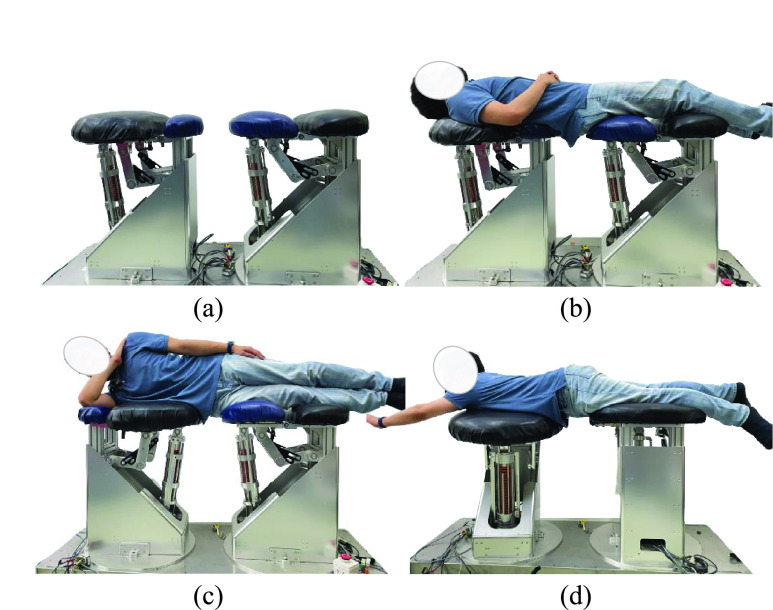
Big three exercise using proposed rehabilitation robot (a) advanced robot (b) Curl-up exercise. (c) Side-bridge exercise (d) Bird-dog exercise.

To verify the exercise effect of the lumbar stabilization exercise using the advanced robot, fifteen healthy adults were recruited as test subjects (age:30 ± 5, height:170 ± 8 cm, weight:70 ± 10 kg). Of the fifteen experimental participants, thirteen participants were newly recruited for this experiment, and the remaining two were participants who had also participated in previous device trials for comparison of the exercise effect between two robots. After attaching five surface EMG sensors to the participant’s five lower back muscles (RA, EO, IO, LE, TE) on the right side of the body unilaterally. The Big three stabilization exercises: curl-up, side-bridge, bird-dog (left hand), bird dog (right hand), a total of four exercises, were performed in the experiment [Fig. 7]. The sEMG sensor adopted the Delsys Trigno wireless system, which can acquire a signal at a 2000 Hz sampling rate. The raw signal obtained in the experiment was sequentially filtered through a band-pass filter (20 to 500 Hz cutting frequency) and RMS filter (200 window size) [20]. Prior to the exercise experiment, the maximum voluntary isometric contraction (MVIC) value was measured in the calisthenics to convert the EMG values between 0 and 100%.

The exercise of the experiment was performed with the calisthenics and the exercise using a robot (assistance rate: 70%, 80%, 90%) of the big three exercises, respectively. The exercise posture was performed for 15 seconds, and the values of the remaining sections excluded (the beginning and end of rehabilitation exercise) were obtained as data. Additionally, to prevent the accumulation of muscle fatigue, a rest period of 60 s was provided between each exercise. The muscle activity data were obtained by performing the same exercise posture three times. To confirm the reduction of the load other than the lower back muscles, after completing all the experimental processes, an additional exercise questionnaire was conducted for the participants. The final purpose of the experiment is to verify whether the stabilization exercise using the robot has a similar muscle activity pattern to that of the calisthenics, and whether the muscle activity monotonically decreases in the same trend by increasing the assist force ratio of the robot.

### B. Results of Clinical Trials Using EMG Sensors

**FIGURE 8.**
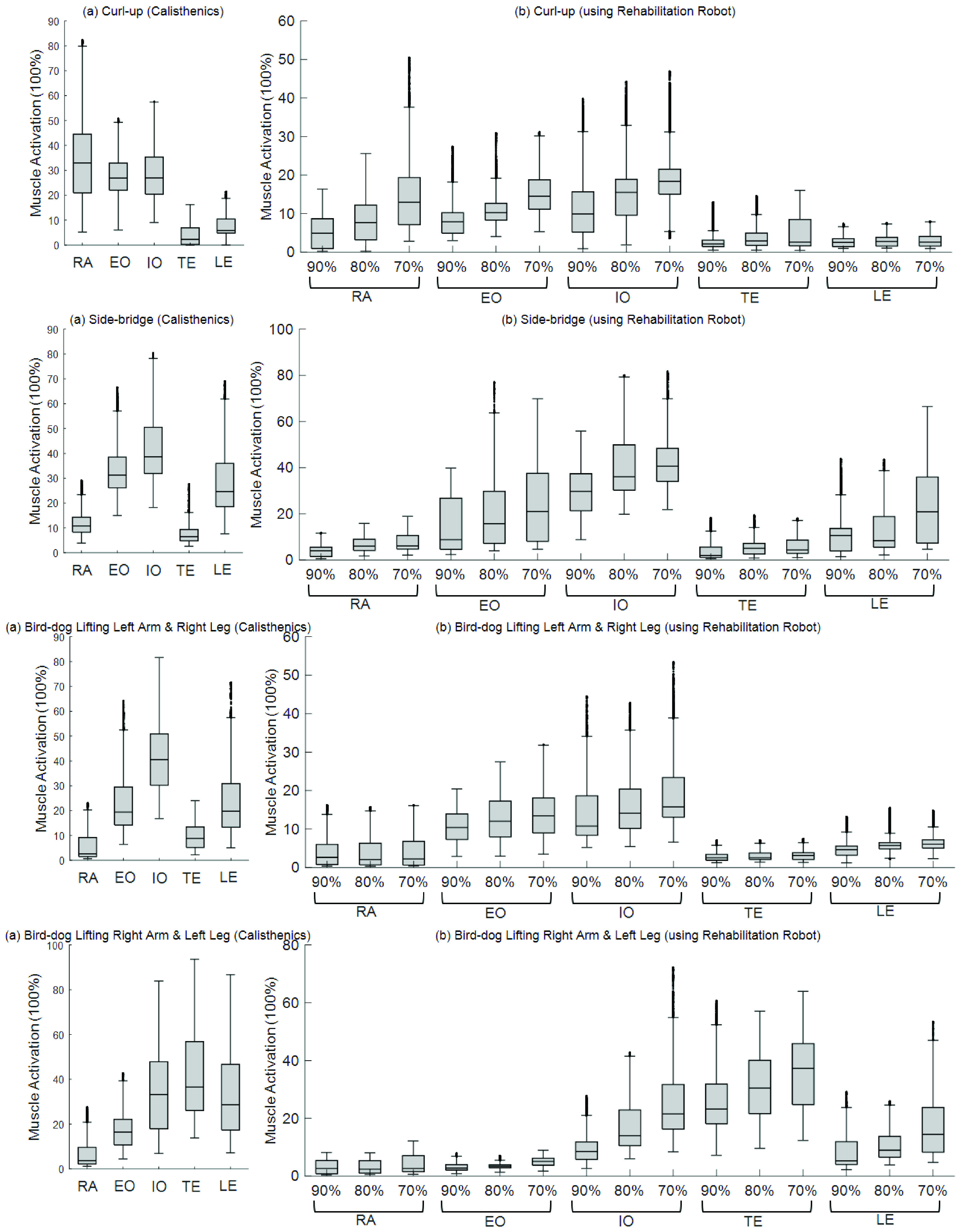
The experiment results of fifteen subjects muscle activation.

The experiment was conducted for fifteen experimental participants, and the experimental results of similar patterns were obtained for all the participants. The experimental results of fifteen participants are shown as a graph [Fig. 8]. The graph shows the bar charts (the median, the lower & upper quartiles, the nonoutlier minimum & maximum, and outliers) of the muscle activity when performing the calisthenics and exercise using the robot according to different assist force rates. The bird-dog exercise is displayed in two graphs because of the asymmetric exercise, which can obtain the same result as measured by 10 sensors by performing the left and right movement, respectively, and acquiring the sEMG sensor data at the same attachment location to the muscle.

From the experiment, the assistance rate decreased in each exercise, and the value of muscle activation for each exercise increased monotonically. Of the total 24 cases excluding the curl-up LE and the left bird-dog TE, 91.4% showed a monotonic increase according to assistance rate decrease, which is a higher monotonic reduction ratio compared to 75% of the previous rehabilitation devices. Additionally, as previously explained about the lumbar stabilization exercise, the curl-up (RA. EO, IO), side-bridge (EO, IO, LE), and bird-dog (EO, IO, TE, LE) muscle groups are activated during the calisthenics. In the experiment using a robot, it was confirmed that a quantitative approach to the same target muscle corresponding to the exercise is possible. Besides, it was observed that the muscle activity was bigger than that of the calisthenics when the assist force rate was low, such as the IO of the side-bridge. This means that when the patient exercised using a robot, the force of the corresponding muscle was used further for balance, thus, it can be corrected while adjusting the assist rate upward accordingly.

For further analysis, it was checked whether the muscles used in the calisthenics and the exercise using the robot were similar by a statistical method. As a result of performing the Kolmogorov-Smirnov test, the muscle activity for each exercise did not follow normality. Therefore, a non-parametric method was used for statistical analysis. First, find the top three muscles based on the median value of the five muscles in each exercise; those three muscles seemed as target training muscles, and remnants seemed as non-training muscles. Then, the Mann-Whitney U test was performed to confirm that these muscles were more significant than the lower two muscles. At this time, all significance was judged on the criteria of p < 0.05.

The muscle that passed the test (p < 0.05) had a significantly higher value than the other two muscles so that it can be defined as a target muscle in the exercise. The median values were used in the exercise using the robot with a 70% assist ratio, which used the lumbar muscles the most in the exercise using the robot. Finally, it was checked whether the muscles used in the calisthenics and the exercise using the robot are the same. The analysis result is in [Table 4].

**TABLE 4:** Muscles Activation Comparison of Calisthenics & Using Rehabilitation Robot

Exercise		Median of the Muscle Activation (%)				
		RA	EO	IO	TE	LE
CU	Calisthenics	32.97*	26.9*	26.97*	2.31	5.82
	$\alpha = 70$%	12.97*	14.49*	18.37*	2.64	2.65
SB	Calisthenics	10.83	31.24*	38.66*	6.55	24.59*
	$\alpha = 70$%	6.11	20.96*	40.57*	4.29	20.8*
BDL	Calisthenics	2.66	19.45*	40.53*	8.85	19.75*
	$\alpha = 70$%	2.21	13.44*	16.73*	3.12	8.01*
BDR	Calistheics	3.64	15.35	33.17*	36.47*	28.63*
	$\alpha = 70$%	4.24	5.05	21.45*	37.32*	14.43*

Bold numbers mean the top three muscles based on the median value of the five muscles in each exercise. Asterisk on the numbers means p is lower than 0.05. For curl-up as an example, as expected, the patterns of muscle activation RA, EO, and IO were significantly greater than those of TE and LE in the calisthenics. The same muscle groups were also used equally in the exercise using the robot. Other exercises showed the same results using the same muscles between the calisthenics and exercise using the robot. Based on this result, it could be seen that the exercise using the robot trained the same muscles as the calisthenics, which means the goal of robot development had been achieved.

However, there were some shortcomings in the experiment. For example, LE of the bird-dog (left) and EO of the bird-dog (right) had lower muscle activation rates compared to calisthenics. From musculoskeletal analysis, The LE muscle is a muscle that controls lateral flexion and trunk extension. The problem is caused by the support point closer to the body than the calisthenics. As an example of the bird-dog exercise, the participant supported the upper body with one arm outstretched and one bent knee on the ground in the calisthenics. In contrast, the participant supported the body with the shoulder and outstretched leg during exercise using the robot, which caused less trunk extension to occur on the body of the participant compared to the calisthenics. The EO of the bird-dog (right) also has similar experiment results. For the same reasons as previously mentioned, the exercise using the robot used one shoulder and the other straight leg as a support point, which caused the joint position of trunk rotation is different from the calisthenics. To further activate those two muscles, giving an extension angle by repositioning the angle of the support point or adjusting the assist force ratio of the robot through various clinical trials could solve the problem. Also, by analyzing these experimental results, the modification of the exercise protocol using robots will result in consistent muscle patterns with the calisthenics.

From the experimental results, it was proved that quantitative load control is possible for the lower back muscles in the lumbar stabilization exercise using a robot, similar to the muscle pattern used in the calisthenics. For the load other than the back muscles, a questionnaire survey was conducted on the participants [Table 5], and it was confirmed that participants perceived the quantitative load adjustment according to assist force ratio and perceived that the load other than the lumbar muscles was reduced for all participants.

**TABLE 5:** Average Score of the Questionaire Survey

Questions (using Robot)	Scoring Method	CU	SB	BD
How difficult to perform Exercise	①Very difficult → ⑤ Very easy	4.1	4.1	4.6
Exercise Simiarities between the Calisthenics	①Very different → ⑤ Very similar	4.1	4.3	4.0
Perception of quantitive changing assist force	①Not recognized → ⑤ Recognized	4.2	4.5	4.3
Perception of the load reduction other than the lumbar muscles	①Not recognized → ⑤ Recognized	4.5	4.5	4.5

### C. Similarity Analysis of Previous & Advanced Robot

**FIGURE 9.**
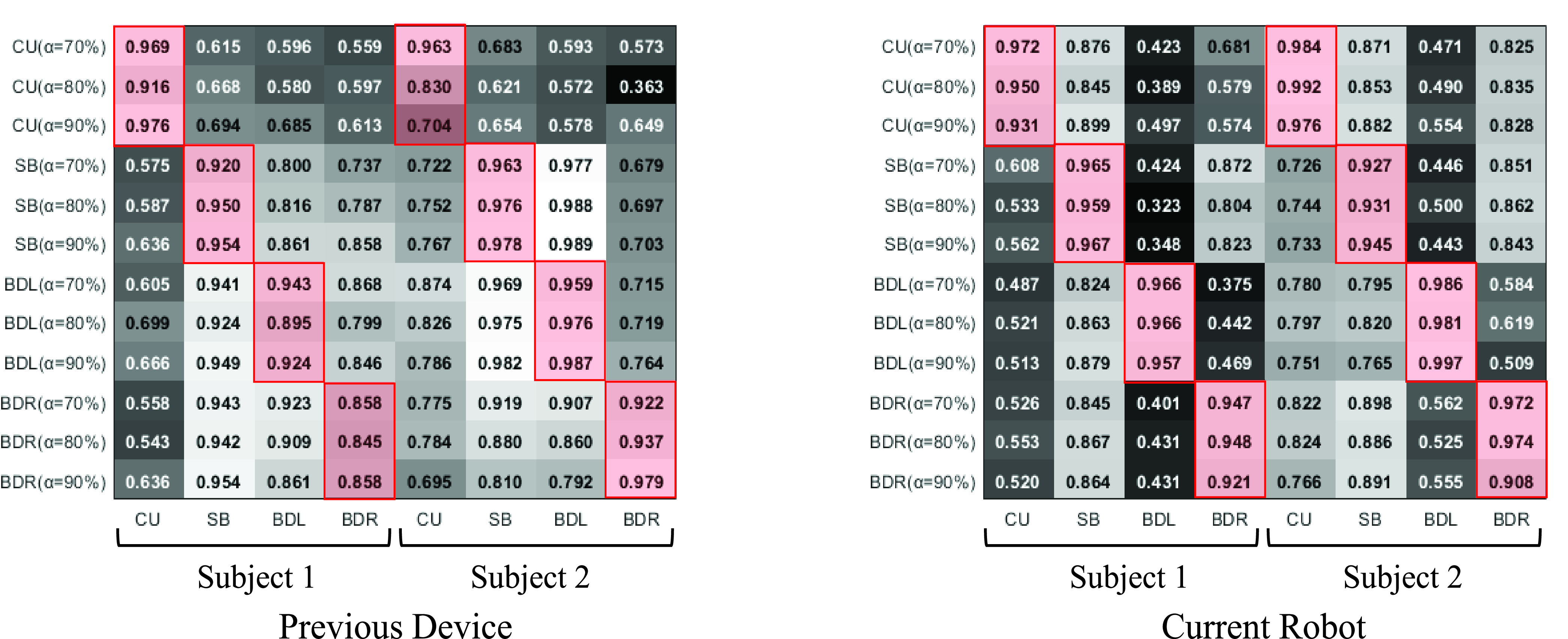
Cosine similarity comparison of the same exercise using previous & current rehabilitation robot with the calistenics (the red box highlighted).

A comparative analysis was also conducted on the results of the experiments performed in the previous device and the advanced robot. Cosine similarity was adopted to verify whether the stabilization exercise performed by the previous device and the advanced robot used the same muscles as the calisthenics. The cosine similarity represents the degree of similarity by dividing the dot product of the vector to be compared with the reference vector by the absolute value of each vector. When the value is closer to 1, the two vectors are assumed to be similar [21]. Here, the vector substituted five muscle activities measured by an sEMG sensor for each exercise. If the value is close to 1, it means that the exercise using the robot is more similar to the muscle use pattern of the calisthenics. For the experimental data, cosine similarity was calculated for the muscle activity data of two persons who participated in the experiment of both devices. The experiment results are shown in the graph [Fig. 9]. The y and x-axes represent the exercises using the robot and calisthenics, respectively. The plot on the left is the cosine similarity to the previous device, whereas that on the right is the similarity result to the advanced robot.

The box highlighted in red is a comparison of the same exercise. For the exercise using the previous device, subject 1 has an overall average of 0.917, and subject 2 has an average of 0.93. The overall average of similarity in the advanced robot is 0.954 and 0.962 for subject 1 and 2, respectively, showing a higher similarity to the calisthenics in the advanced robot.

Comparing the similarity between the two robots for each exercise, subject 1’s side-bridge was increased from 0.941 to 0.964, the bird-dog (left) increased from 0.921 to 0.963, and the bird-dog (right) increased from 0.854 to 0.939. Subject 2 also was increased in the curl-up from 0.832 to 0.984, the bird-dog (left) increased from 0.974 to 0.988, and the bird-dog (right) increased from 0.946 to 0.951. In some cases, the degree of similarity decreased slightly. Subject 1’s curl-up was slightly decreased from 0.954 to 0.951, whereas subject 2’s side-bridge was decreased from 0.972 to 0.934. Particularly, it was recognized as a problem resulting from relatively low muscle activation similarity between the calisthenics and the exercise using the previous device for the bird-dog posture; the bird-dog (right) of subject 1 was an average of 0.853 in the previous device. The average of subject 1 was improved to 0.939 in the advanced robot, which can be confirmed from the results that the similarity is increased.

Experiment results mean that the goal of this study is achieved. The goal is to arrange the support points of the device and the acting points of the force of the SEA similarly to the calisthenics through advanced mechanical design so that the muscles activated pattern in the exercise using the robot and the calisthenics are similar.

## IV. Conclusion and Future Works

This study proposed a rehabilitation robot that can compensate for the shortcoming of the lumbar stabilization exercise, which makes it difficult for patients to perform the exercise efficiently owing to the load on the lower back and the loads on the other part of the body. The device was designed to satisfy two features so that the patient can perform the exercise safely. First, it was designed to quantitatively control the load on the back muscles to be trained. Second, we aimed to lower loads other than the back muscles to be trained in the exercise using the robot.

As a previous study, a bed-type rehabilitation device using a series elastic actuator was proposed for clinical needs. By experimenting with 20 people, some exercises exhibited similarities with the calisthenics; however, a low similarity result of the muscle activation in specific exercises was observed. Additionally, a number of difficulties in balancing were observed in the experiment using the device, which was a problem that occurred because the design of the device was not appropriate for ergonomic design and clinical analysis. This problem limits the effectiveness of the exercise and addressing clinical need.

In this study, for advanced solutions to a clinical need with experiment results and healthcare relevance, the mechanical design of the robotic system excluding the actuator was redesigned for the stabilization exercise through the feedback received from the subjects and the preceding experiment, and the static analysis of the stabilization exercise was performed. To reduce the impedance that the subject feels when exercising using a robot, a Cascade controller was applied, which is robust against disturbance and friction also capable of accurately following the desired force and implementing a low-impedance. From the examination of fifteen participants that participated in the previous device experiment, it was proved that it is possible to adjust the quantitative load on the target lumbar muscle to be trained in the lumbar stabilization exercise using the advanced robot. The muscle pattern showed a higher similarity compared to the previous device. In particular, for the bird-dog posture, which showed low muscle activity similarity in the previous device, a higher similarity was obtained in the advanced robot through an experiment. This proves that the support and acting points of the force provided by the advanced robot are properly set compared to the previous device. It is shown through the experimental results that it is possible to finely control the load and training the desired muscle by adequately placing the support and assist force point.

As future works, It is expected to enter the clinical phase in the near future. If it passes the clinical stage, an experiment on actual lumbar patients and the elderly will be conducted and derive clinical results. As a follow-up study, a full-body dynamic model including the lower back could be constructed. Through dynamic simulation, it is expected that accurate access to specific muscles by diversifying support points and assist force points will be possible. Additionally, because it is possible to access specific muscles to be trained through the robot, a new rehabilitation exercise protocol using robots can be developed. Furthermore, the currently developed rehabilitation robot is bulky and heavy because of the bed type device, thus, there is a limit to its mobility and popularization. Based on the existing lumbar research, the development of a portable type lumbar rehabilitation robot or wearable robot-type lumbar rehabilitation robot is also expected.
